# Oxidative and endoplasmic reticulum stresses are involved in palmitic acid-induced H9c2 cell apoptosis

**DOI:** 10.1042/BSR20190225

**Published:** 2019-05-21

**Authors:** Lei Yang, Gaopeng Guan, Lanjie Lei, Jianyun Liu, Lingling Cao, Xiangguo Wang

**Affiliations:** 1 Beijing Key Laboratory of Traditional Chinese Veterinary Medicine, Animal Science and Technology College, Beijing University of Agriculture, Beijing 102206, China; 2 College of Basic Medical Science, Jiujiang University, Jiujiang, Jiangxi 332000, China; 3 Affiliated Hospital of Jiujiang University, Jiujiang University, Jiujiang, Jiangxi 332000, China; 4 Key Laboratory of System Bio-medicine of Jiangxi Province, Jiujiang University, Jiujiang, Jiangxi 332000, China; 5 Department of Endocrinology, The First Hospital of Jiujiang City, Jiujiang 332000, China

**Keywords:** apoptosis, endoplasmic reticulum stress, H9c2 cells, oxidative stress, palmitic acid

## Abstract

Palmitic acid (PA) is the most common saturated long-chain fatty acid that causes damage to heart muscle cells. However, the molecular mechanism of PA toxicity in myocardial cells is not fully understood. In the present study, we explored the effects of PA on proliferation and apoptosis of H9c2 cardiomyocytes, and uncovered the signaling pathways involved in PA toxicity. Our study revealed induction of both oxidative and endoplasmic reticulum (ER) stresses and exacerbation of apoptosis in PA-treated H9c2 cells. Inhibition of oxidative stress by N-acetylcysteine (NAC) reduced apoptosis and decreased ER stress in PA-treated H9c2 cells. Moreover, inhibition of ER stress by 4-phenyl butyric acid decreased apoptosis and attenuated oxidative stress. In summary, the present study demonstrated that oxidative stress coordinates with ER stress to play important roles in PA-induced H9c2 cell apoptosis.

## Introduction

Diets rich in high fat foods, especially saturated fats, cause obesity, leading to serious health problems, such as type 2 diabetes and lipotoxic cardiomyopathy [1]. It has been confirmed that the degree of lipid accumulation is linked to cardiac dysfunction of human diabetic hearts [2,3]. Palmitic acid (PA), the most common saturated long-chain fatty acid, triggers apoptosis in many cell types including cardiomyocytes [4]. Cardiomyocyte apoptosis leads to myocardial injury and ultimately results in heart dysfunction to some extent [5]. Previous studies have revealed the mechanisms of lipotoxicity in diabetic cardiomyopathy, including endoplasmic reticulum (ER) stress [6] and oxidative stress [7].

The ER is an indispensable and elaborate eukaryotic organelle that is primarily responsible for synthesis, packaging, and assembly of secretory and membrane proteins [8]. Any physiological or pathological perturbation can disrupt ER homeostasis and cause accumulation of unfolded or misfolded proteins in the ER lumen, resulting in ER stress [9]. Glucose-regulated protein 78 (GRP78) and CCAAT/enhancer binding protein homologous protein (CHOP) are both activated by the ER stress response [8]. GRP78 interacts with activating transcription factor 6 (ATF6), inositol requiring enzyme 1α (IRE1α), and pancreatic ER kinase (PERK) in the ER membrane, which maintains these transmembrane proteins in their inactive configuration. When the unfolded protein response fails to manage misfolded and unfolded proteins, the stress ultimately triggers apoptosis [9].

Oxidative stress plays a critical role in the pathogenesis of diabetic cardiomyopathy, which might impair antioxidant defense systems [10]. Reactive oxygen species (ROS), which are produced in all cellular compartments as a result of exposure to toxic agents and natural byproducts of mitochondrial respiration, are a particularly destructive aspect of oxidative stress [11]. NADPH oxidase 2 (NOX2) present in cardiovascular tissues is a well-characterized superoxide-generating enzyme [12]. Numerous studies have suggested that increased ROS activate various signaling pathways, resulting in DNA damage and apoptosis [13,14]. Previous studies have also shown that oxidative and ER stresses are closely related [15,16]. Both oxidative stress [21] and ER stress [9] are involved in PA-induced apoptosis. However, there is little known about the relationship between oxidative and ER stresses in PA-induced H9c2 cell apoptosis.

In the present study, PA-treated H9c2 cells were used as a model to examine the molecular mechanism of lipotoxic cardiomyopathy. Here, we investigated whether oxidative and ER stress pathways are involved in H9c2 cell apoptosis and explored the relationship between oxidative and ER stresses in PA-induced H9c2 cell apoptosis.

## Materials and methods

### Materials

N-acetylcysteine (NAC; antioxidant), PA, 4-phenylbutyrate (4-PBA; ER stress inhibitor), and bovine serum albumin (BSA) were purchased from Sigma–Aldrich (St. Louis, MO, U.S.A.). Fetal bovine serum (FBS) was purchased from Gibco (Grand Island, NY, U.S.A.). Dulbecco’s modified Eagle’s medium (DMEM) was obtained from HyClone (Logan, Utah, U.S.A.). H9c2 cardiomyocytes were purchased from the Chinese Academy of Sciences (Shanghai, China). A caspase 3 Activity Assay Kit was purchased from the NanJing JianCheng Bioengineering Institute (Nanjing, Jiangsu, China). NaOH, phosphate-buffered saline (PBS), penicillin, and gentamicin were obtained from Solarbio (Beijing, China). A CCK8 Assay Kit, ROS Assay Kit, and Annexin V-PE/PI Apoptosis Analysis Kit were purchased from the Beyotime Institute of Biotechnology (Shanghai, China). TRIzol and a PrimeScript® RT Reagent Kit were purchased from Invitrogen (Carlsbad, CA, U.S.A.). PVDF membranes were obtained from Millipore (Bedford, MA).

### PA preparation

PA was prepared by soaping palmitate with sodium hydroxide and mixing with BSA. PA (20 mM in 0.01 M NaOH) was incubated at 70°C for 30 min. Then, the fatty acid soaps were complexed with 5% fatty acid-free BSA in PBS in a 1:3 volume ratio to produce a 5 mM PA stock solution, which was stored at −20°C. Before application, the stock solution was diluted in culture medium.

### Cell culture and treatments

H9c2 cells were cultured in DMEM supplemented with 10% FBS, 50 μg/ml penicillin, and 50 μg/ml gentamicin in a humidified incubator at 37°C with 5% CO_2_. The growth medium was changed every 3 days. When the cells reached 70–80% confluence, they were treated with 100–800 μM PA for 24 h. In other experiments, H9c2 cells were exposed to 400 µM PA in the presence or absence of 500 nM 4-PBA and 2 mM NAC. After incubation for 24 h, the cells were collected to assess cell viability, apoptosis, caspase 3 activity, and B-cell lymphoma 2 (BCL-2)-associated X protein (BAX), GRP78, CHOP, and NOX2 expression.

### Measurement of cell viability

H9c2 cell proliferation was monitored by a CCK8 assay, in accordance with the manufacturer’s instructions. Cells were plated at 2 × 10^4^ per well in 96-well plates. After treatment, 10 μl CCK8 was added to each well, and the cells were incubated for 2 h at 37°C. The number of viable cells was measured by a microplate reader (Bio-Rad 680; Bio-Rad, Hercules, CA, U.S.A.) at 450 nm.

### Apoptosis measurement

Apoptosis was determined using the Annexin V-PE/PI Apoptosis Analysis Kit. After treatments, the cells were collected and resuspended in 500 μl binding buffer. After incubation with 10 μl Annexin V-PE and 5 μl PI for 15 min at 25°C in the dark, the apoptotic rates of the cells were detected by a flow cytometer (Becton, Dickinson and Company, U.S.A.). Early apoptotic cells were Annexin V+/PI− cells, late apoptotic cells were Annexin V+/PI+ cells, necrotic cells were Annexin V−/PI+ cells, and Annexin V−/PI− cells were considered as live cells.

### ROS measurement

ROS measurement was carried out in accordance with the procedure of the ROS Assay Kit. After treatments, the cells were incubated with 10 µM DCFH-DA at 37°C for 20 min. Then, the cells were washed three times, and staining with the ROS-sensitive dye was assessed under an inverted fluorescence microscope (Olympus, Japan) at an excitation wavelength of 488 nm and emission wavelength of 525 nm. Fluorescence was examined by image analysis software ImageJ (Version 1.49).

### Caspase 3 activity measurement

Caspase 3 activity was measured using a caspase 3 Activity Colorimetric Assay Kit. After treatments, cells were harvested by scraping, collected by centrifugation, and incubated in lysis buffer on ice for 15 min. Then, the lysate was centrifuged at 15000 rpm for 15 min at 4°C, and the protein content was determined, after which the caspase 3 substrate was measured using the microplate reader at 405 nm.

### RNA isolation and qRT-PCR

After treatments, cells were collected and lysed with TRIzol reagent. Total RNA was reverse transcribed using the PrimeScript® RT Reagent Kit, in accordance with the manufacturer’s instructions. The qRT-PCR procedure was carried out using a Bio-Rad IQ5 and Bio-Rad IQ5 Optical System Software (Bio-Rad). The PCR cycling conditions were 1 cycle of 30 s at 95°C, followed by 40 cycles of 5 s at 95°C and 30 s at 60°C. Primers are listed in Supplementary Table S1. β-Actin served as a reference gene. mRNA expression of oxidative stress and ER stress markers after the treatments is shown in Supplementary Figures S1 and S2.

### Western blotting

After treatments, cells were collected, washed with ice-cold PBS, and lysed with RIPA buffer. The total protein concentration was then measured by the BCA assay. Total protein (50 µg) in each sample was loaded into each well of a 12% SDS/PAGE gel and separated by electrophoresis. Proteins were then transferred on to PVDF membranes. After blocking in TBST with 10% dry nonfat milk for 2 h, the membranes were incubated with a primary antibody (Supplementary Table S2) overnight at 4°C. After washing, the membranes were incubated with a secondary antibody conjugated to horseradish peroxidase at 37°C for 30 min. Immunoreactive bands were visualized using a Super Signal West Pico kit, in accordance with the manufacturer’s instructions. Protein band densities were semi-quantitated by densitometric analysis using ImageJ (Version 1.49).

### Statistical analyses

All experiments were repeated at least three times for each group. Data are presented as the mean  ±  S.E.M. Results were analyzed by ANOVA, followed by Fisher’s least significant difference test and the independent samples Student’s *t* test with SPSS software, version 13.0 (SPSS, Chicago, IL, U.S.A.).

## Results

### Effects of PA on proliferation and oxidative stress in H9c2 cells

**Figure 1:**
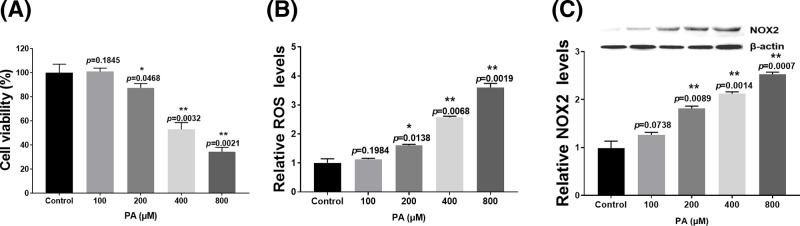
Effect of PA on proliferation and oxidative stress of H9c2 cells (**A**) Cells were treated with various concentrations (100–800 µM) of PA for 24 h and then processed for cell viability analysis. (**B**) Cells were treated with various concentrations (100–800 µM) of PA for 24 h and then processed for the ROS assay. (**C**) Cells were treated with various concentrations (100–800 µM) of PA for 24 h and then analyzed for NOX2 expression. NOX2 expression was normalized to β-actin levels. Data are presented as the mean ± S.E.M. of three independent experiments, *P<0.05, **P<0.01 versus the control group.

To analyze the effect of PA on the proliferation and ROS generation of H9c2 cells, the cells were treated with 100–800 µM PA for 24 h. The results showed that PA decreased cell viability in a dose-dependent manner from 200 to 800 µM concentrations compared with the control group, and the IC_50_ value was approximately 400 µM (Figure 1A, *P*=0.0032). In addition, PA induced ROS generation (Figure 1B) and NOX2 expression (Figure 1C and Supplementary Figure S1a) in H9c2 cells in a dose-dependent manner from 200 to 800 µM concentrations compared with the control group. These results showed that PA reduces cell viability and promotes oxidative stress in H9c2 cells.

### Effects of PA on apoptosis-related gene expression in H9c2 cells

**Figure 2:**
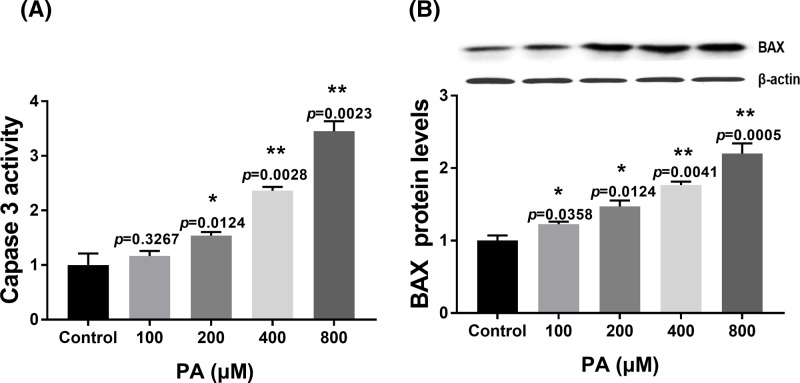
Effect of PA on apoptosis-related protein expression in H9c2 cells (**A**) Caspase 3 activity in H9c2 cells after treatment with various doses of PA (100–800 µM) for 24 h. (**B**) Relative BAX expression in H9c2 cells after treatment with various doses of PA (100–800 µM) for 24 h. BAX expression was normalized to β-actin levels. Data are presented as the mean ± S.E.M. of three independent experiments, *P<0.05, **P<0.01 versus the control group.

To explore the mechanism of PA in apoptosis of H9c2 cells, apoptosis-related gene expression (caspase 3 and BAX) was measured by a colorimetric assay and Western blotting. PA enhanced the levels of caspase 3 activity in a dose-dependent manner from 200 to 800 µM concentrations compared with the control group at 24 h (Figure 2A). In addition, PA induced BAX protein in expression in a dose-dependent manner from 100 to 800 µM concentrations compared with the control group at 24 h (Figure 2B). These results indicated that PA-induced H9c2 cell apoptosis was related to caspase 3 activity and BAX.

### Effect of PA on ER stress in H9c2 cells

**Figure 3:**
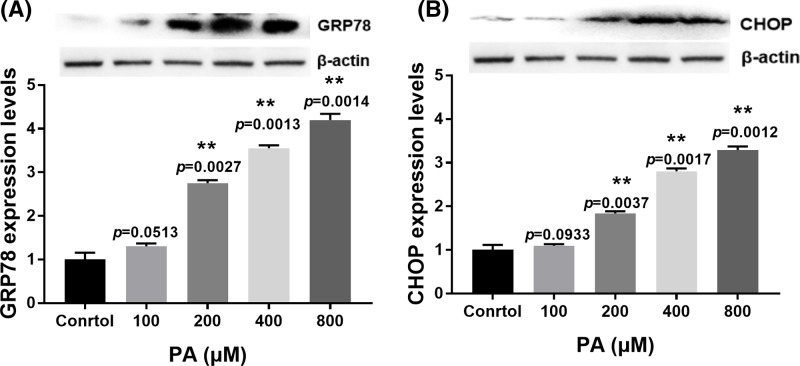
Effect of PA on ER stress-related protein expression in H9c2 cells (**A**) Relative expression of GRP78 in H9c2 cells after treatment with various doses of PA (100–800 µM) for 24 h. (**B**) Relative expression of CHOP after treatment with various doses of PA (100–800 µM) for 24 h. Protein expression was normalized to β-actin levels. Data are presented as the mean ± S.E.M. of three independent experiments, 0.05<**P<0.01 versus the control group.

To investigate whether ER stress is involved in PA-induced apoptosis, we determined the expression of ER stress marker proteins GRP78 and CHOP. Our results showed that ER stress was active and expression of CHOP and GRP78 was up-regulated at mRNA and proteins levels in a dose-dependent manner from 200 to 800 µM concentrations compared with the control group at 24 h (Figures 3A,B and Supplementray Figure S1b,c). These results indicated that PA induced ER stress during H9c2 cell apoptosis.

### Role of oxidative stress in PA-induced apoptosis and ER stress in H9c2 cells

**Figure 4:**
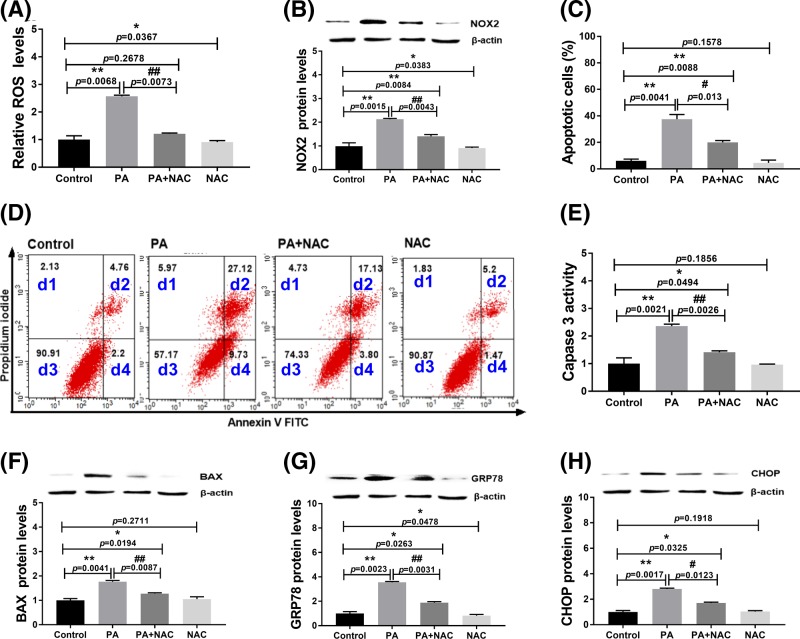
Effect of NAC on PA-induced apoptosis and ER stress of H9c2 cells at 24 h (**A**) ROS levels in H9c2 cells after treatments; (**B**) NOX2 expression levels in H9c2 cells after treatments. (**C,D**) Apoptosis analysis by flow cytometry. d1: necrotic cells; d2: late apoptotic cells; d3: live cells; d4: early apoptotic cells. (**E**) Caspase 3 activity in H9c2 cells after treatments; (**F**) BAX expression levels in H9c2 cells after treatments. (**G**) GRP78 expression levels in H9c2 cells after treatments; (**H**) CHOP expression levels in H9c2 cells after treatments. Protein expression levels were normalized to β-actin levels. PA: 400 µM palmitic acid; NAC: 2 mM N-acetylcysteine. Data are presented as the mean ± S.E.M. of three independent experiments, *P<0.05, **P<0.01 versus the control group and ^#^*P*<0.05, ^##^*P*<0.01 represent PA+NAC treated group versus the PA-treated group.

To confirm the role of oxidative stress in apoptosis and ER stress of PA-treated H9c2 cells, we used NAC to inhibit oxidative stress during the treatments. NAC is a source of sulfhydryl groups in cells and acts as a free radical scavenger. The results indicated that NAC treatment completely reversed the generation of ROS (Figure 4A, *P*=0.2678) and partially reversed the expression of NOX2 caused by PA (Figure 4B and Supplementary Figure S2a). Furthermore, NAC effectively suppressed apoptosis (Figure 4C,D) and expression of caspase 3 and BAX induced by PA (Figure 4E,F). These results indicated that oxidative stress is involved in PA-induced cell apoptosis. Additionally, NAC treatment significantly decreased the expression of GRP78 and CHOP induced by PA in H9c2 cells (Figure 4G,H and Supplementary Figure S2b,c), suggesting that NAC treatment significantly alleviated PA-induced ER stress. These data indicated that oxidative stress is involved in PA-induced ER stress and apoptosis.

### Roles of ER stress in PA-induced apoptosis and oxidative stress of H9c2 cells

**Figure 5:**
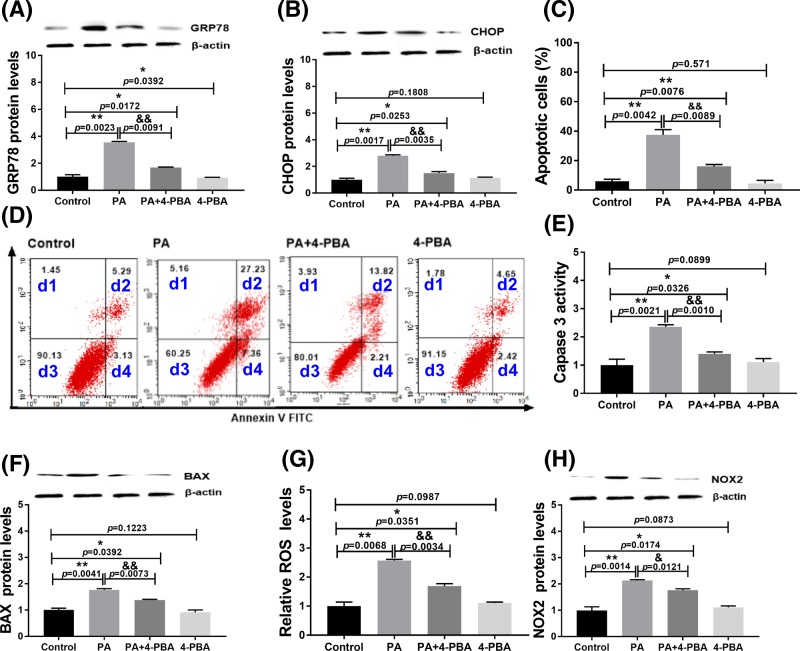
Effect of 4-PBA on PA-induced apoptosis and oxidative stress of H9c2 cells at 24 h (**A**) GRP78 expression levels in H9c2 cells after treatments. (**B**) CHOP expression levels in H9c2 cells after treatments. (**C,D**) Apoptosis analysis by flow cytometry. d1: necrotic cells; d2: late apoptotic cells; d3: live cells; d4: early apoptotic cells. (**E**) Caspase 3 activity in H9c2 cells after treatments. (**F**) BAX expression levels in H9c2 cells after treatments. (**G**) ROS levels in H9c2 cells after treatments. (**H**) NOX2 expression levels in H9c2 cells after treatments. Protein expression levels were normalized to β-actin levels. PA: 400 µM palmitic acid; 4-PBA: 500 nM 4-PBA. Data are presented as the mean ± S.E.M. of three independent experiments, *P<0.05, **P<0.01 versus the control group and ^&^*P*<0.05, ^&&^*P*<0.01 represent PA+4-PBA treated group versus the PA-treated group.

To further confirm the role of ER stress and its relationship with oxidative stress in PA-induced cell apoptosis, we used 4-PBA to inhibit ER stress during the treatments. 4-PBA, an ER stress inhibitor, can be used to promote protein folding and prevent aggregation of misfolded proteins. The results showed that 4-PBA partially suppressed ER stress-related protein expression (GRP78 and CHOP) induced by PA (Figure 5A,B and Supplementary Figure S2b,c). Moreover, treatment with 4-PBA decreased the apoptosis rate (Figure 5C,D) and expression of proapoptotic factors (caspase 3 and BAX; Figure 5E,F). These results indicated that ER stress is involved in PA-induced H9c2 cell apoptosis. Additionally, 4-PBA partially inhibited the ROS generation and NOX2 expression induced by PA (Figure 5G,H and Supplementary Figure S2a). These data indicated that ER stress is related to oxidative stress in PA-induced H9c2 cell apoptosis.

## Discussion

In the present study, we examined the mechanism of saturated fatty acid-induced cardiomyocyte injury. To this end, we used PA to investigate the mechanisms of lipotoxicity in H9c2 cells. We first evaluated the effect of PA on the proliferation of H9c2 cells. Our data showed that PA reduced cell viability, supporting previous studies showing that PA inhibits cell proliferation and induces apoptosis in many cell types and cell lines, such as neuronal cells [17], stem cells [18], and hepatocytes [19].

Oxidative stress plays a major role in the pathogenesis of diabetic cardiomyopathy [10]. Studies have also reported that oxidative stress is involved in apoptosis [13,14]. In the current study, we found that treatment of H9c2 cells with PA increased ROS generation and NOX2 expression in a dose-dependent manner. The up-regulated NOX2 enzyme contributes to oxidative stress and cardiovascular disease [20]. Consistent with previous studies in other cell types [21], we also found that PA promoted ROS generation in H9c2 cells, suggesting that oxidative stress may be one of the reasons for PA-induced H9c2 cell apoptosis.

Next, we detected expression of apoptosis-related genes, including BAX and caspase 3, during PA-induced H9c2 cell apoptosis. Caspase 3 is processed into cleaved caspase 3 in the early steps of apoptosis, and expression of caspase 3 positively correlates with the rate of apoptosis [9]. BCL-2 is an important protein that promotes cellular survival and inhibits the actions of proapoptotic proteins. Moreover, BAX has a very important proapoptotic effects among BCL-2 family members [9]. Our study showed that PA induced high levels of BAX and caspase 3 as reported previously [22], suggesting that PA regulates caspase 3 activation and the BCL-2/BAX pathway to induce apoptosis in H9c2 cells.

ER stress is a cellular rescue mechanism that is activated to ease stress during various pathophysiological insults. However, continuous and excess ER stress pathway activation can result in apoptosis [8]. Previous studies have confirmed that GRP78 and CHOP are ER stress markers [9]. In our study, we found that PA induced GRP78 and CHOP expression. These results were consistent with a recent study showing increased GRP78 and CHOP during PA-induced myocardial apoptosis *in vitro* and *in vivo* [22]. Taken together, these data suggest that the ER stress pathway is active in PA-treated H9c2 cells.

To understand the role of oxidative stress in PA-mediated H9c2 cell apoptosis, we suppressed oxidative stress by NAC treatment. The results showed that NAC dramatically decreased the concentrations of ROS. Moreover, GRP78 and CHOP were significantly decreased after NAC treatment. In addition, NAC partially reversed PA-induced cell apoptosis and the decrease in cell viability. These results were consistent with previous studies showing that PA induces oxidative stress and apoptosis in pancreatic β-cells [23]. Our study revealed for the first time that oxidative stress is involved in PA-induced H9c2 cell apoptosis. These results further support previous studies showing that oxidative stress is related to H9c2 cell apoptosis during ischemia/reperfusion injury [24].

To analyze the role of ER stress in PA-mediated H9c2 cell apoptosis, we suppressed ER stress by 4-PBA treatment. We found that inhibition of ER stress by 4-PBA obviously rescued PA-triggered cell apoptosis, the decrease in cell viability, and expression of GRP78 and CHOP in H9c2 cells. Our data further support a previous study showing that PA induces apoptosis in primary cardiomyocytes via ER stress [22]. This is the first report indicating that ER stress is involved in PA-induced apoptosis of H9c2 cells and revealed the role of ER stress in apoptosis of another cell type.

Previous studies have shown the association of oxidative and ER stresses with apoptosis [15,16]. Our study demonstrated that both ER and oxidative stresses were involved in PA-induced H9c2 cell apoptosis. Therefore, we further analyzed the potential relationships of oxidative and ER stresses in PA-induced apoptosis. We found that inhibition of oxidative stress by NAC partially blocked ER stress-related protein expression. In addition, NAC altered PA-induced apoptosis and related protein expression. These findings indicated that oxidative stress was an inducer of ER stress in PA-induced H9c2 cell apoptosis.

Next, we investigated the effects of ER stress on ROS generation. Inhibition of ER stress by 4-PBA significantly decreased ROS generation and NOX2 expression. These results suggested that ER stress is one of the causes of oxidative stress in PA-induced H9c2 cell apoptosis. Moreover, blocking oxidative stress by NAC decreased ER stress, suggesting that ROS generation was an upstream factor in PA-induced H9c2 cell apoptosis. Conversely, blocking ER stress with 4-PBA significantly decreased oxidative stress. These results indicated that oxidative and ER stresses interact with each other during PA-induced cell apoptosis. The possible mechanism may be that oxidative stress disrupts ER homeostasis and causes ER stress. Therefore, inhibition of oxidative stress suppresses ER stress during PA treatment. In addition, persistent ER stress may cause mitochondrial dysfunction that further induces oxidative stress. Thus, inhibition of ER stress can also inhibit oxidative stress. However, the exact underlying mechanism requires further investigation.

In summary, our study demonstrates that both oxidative and ER stresses are involved in PA-induced H9c2 cell apoptosis, and there is a cross-talk between oxidative and ER stresses during this process. The present study offers new insights into the molecular mechanisms of lipotoxicity in diabetic cardiomyopathy.

## Supporting information

**Supplementary Figure S1:** 

**Supplementary Figure S2:** 

**Supplemental Table S1:** Primer sequences used for RT-qPCR.

**Supplemental Table S2:** Antibody used in this study.

## Abbreviations

BAX: B-cell lymphoma 2-associated X protein
BCL-2: B-cell lymphoma 2
BSA: bovine serum albumin
CHOP: CCAAT/enhancer binding protein homologous protein
DMEM: Dulbecco’s modified Eagle’s medium
ER: endoplasmic reticulum
FBS: fetal bovine serum
GRP78: glucose-regulated protein 78
NAC: N-acetylcysteine
NOX2: NADPH oxidase 2
PA: palmitic acid
PBS: phosphate-buffered saline
ROS: reactive oxygen species
4-PBA: 4-phenylbutyrate
