# Supplementary material for: Oxidative and endoplasmic reticulum stresses are involved in palmitic acid-induced H9c2 cell apoptosis
Source: Biosci Rep. 2019 May 21;39(5):BSR20190225. doi: 10.1042/BSR20190225 (PMC6527925; doi:10.1042/BSR20190225)
Supplement: Supplementary file 1 [file bsr20190225_Supp1.pdf]

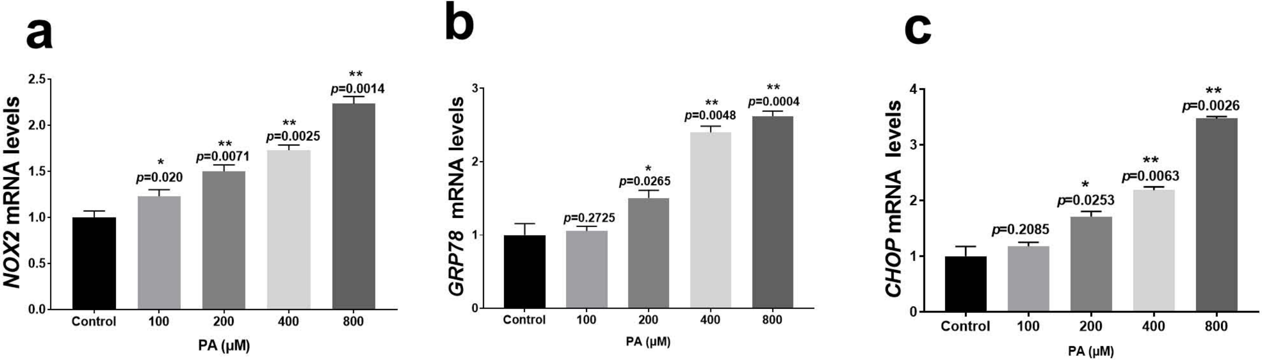

a
NOX2 mRNA levels
0.0
0.5
1.0
1.5
2.0
2.5
*
p=0.020
**
p=0.0071
**
p=0.0025
**
p=0.0014
Control
100
200
400
800
PA (μM)
b
GRP78 mRNA levels
0
1
2
3
p=0.2725
*
p=0.0265
**
p=0.0048
**
p=0.0004
Control
100
200
400
800
PA (μM)
c
CHOP mRNA levels
0
1
2
3
4
p=0.2085
*
p=0.0253
**
p=0.0063
**
p=0.0026
Control
100
200
400
800
PA (μM)

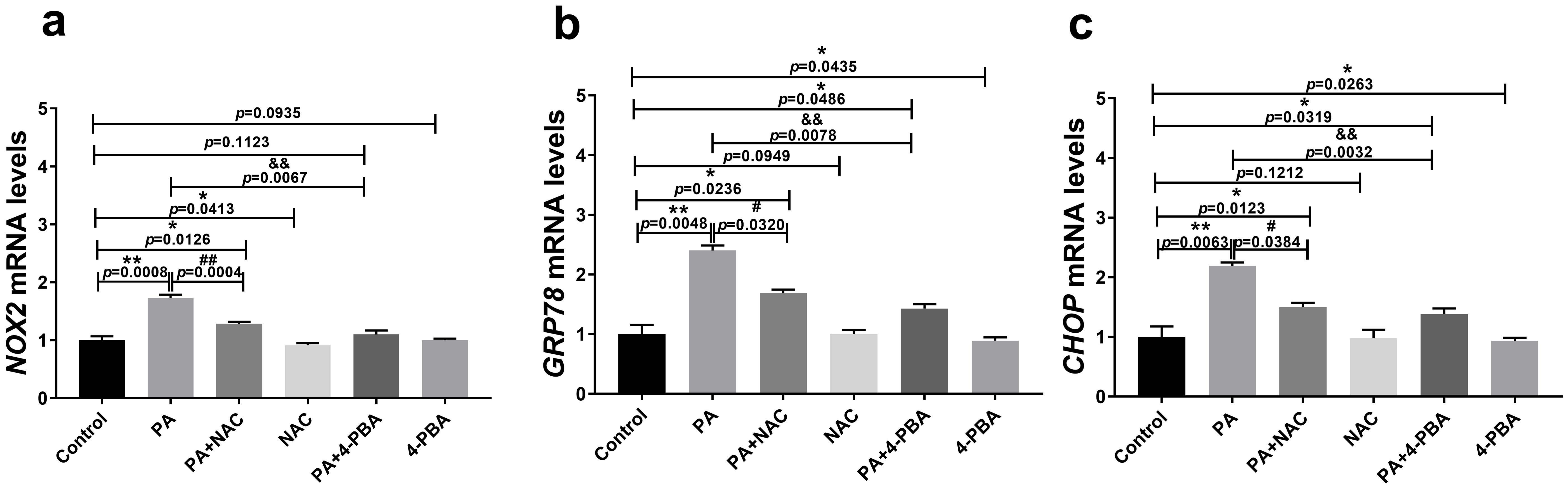
a
NOX2 mRNA levels
p=0.0935
p=0.1123
&&
p=0.0067
*
p=0.0413
*
p=0.0126
**
##
p=0.0008
p=0.0004
Control
PA
PA+NAC
NAC
PA+4-PBA
4-PBA
b
GRP78 mRNA levels
*
p=0.0435
*
p=0.0486
&&
p=0.0078
p=0.0949
*
p=0.0236
**
#
p=0.0048
p=0.0320
c
CHOP mRNA levels
*
p=0.0263
*
p=0.0319
&&
p=0.0032
p=0.1212
*
p=0.0123
**
#
p=0.0063
p=0.0384

Table S1. Primer sequences used for RT-qPCR.

Gene	Forward (5′-3′)	Reverse (5′-3′)	Tm(°C)
NOX2	CGGGATAGTTGACTTCACCATCC	ATTCTGGTGTTGGGGTGTTGACT	60
GRP78	TGGGTCGACTCGAATTCCAAAG	GTCAGGCGATTCTGGTCATTGG	60
CHOP	AATCAGAGCTGGAACCTGAGGA	TGCTTTCAGGTGTGGTGATGTATG	60
β-actin	GTTTGAGACCTTCAACACCCCC	GTGGCCATCTCTCTTGCTCGAAGTC	60

Table S2 Antibody used in this study

**Target(diluted)**	**Catalogue number**	**Company**
NOX2(1:1000)	19013-1-AP	Proteintech
β-actin(1:2000)	sc-47724	Santa Cruz
BAX(1:500)	sc-4239	Santa Cruz
CHOP(1:1000)	ab10444	Abcam
GRP78(1:1000)	ab32618	Abcam
